# Application of Sequence-Dependent Electrophoresis Fingerprinting in Exploring Biodiversity and Population Dynamics of Human Intestinal Microbiota: What Can Be Revealed?

**DOI:** 10.1155/2008/597603

**Published:** 2008-12-14

**Authors:** Geert Huys, Tom Vanhoutte, Peter Vandamme

**Affiliations:** Laboratory of Microbiology, Faculty of Sciences, Ghent University, K.L. Ledeganckstraat 35, 9000 Ghent, Belgium

## Abstract

Sequence-dependent electrophoresis (SDE) fingerprinting techniques such as denaturing gradient gel electrophoresis (DGGE) have become commonplace in the field of molecular microbial ecology. The success of the SDE technology lays in the fact that it allows visualization of the predominant members of complex microbial ecosystems independent of their culturability and without prior knowledge on the complexity and diversity of the ecosystem. Mainly using the prokaryotic 16S rRNA gene as PCR amplification target, SDE-based community fingerprinting turned into one of the leading molecular tools to unravel the diversity and population dynamics of human intestinal microbiota. The first part of this review covers the methodological concept of SDE fingerprinting and the technical hurdles for analyzing intestinal samples. Subsequently, the current state-of-the-art of DGGE and related techniques to analyze human intestinal microbiota from healthy individuals and from patients with intestinal disorders is surveyed. In addition, the applicability of SDE analysis to monitor intestinal population changes upon nutritional or therapeutic interventions is critically evaluated.

## 1. INTRODUCTION

The mammalian intestinal tract comprises a highly complex population of microorganisms reaching up to 10^14^ bacteria in the large intestine [1]. Starting off as a sterile system at birth, microbial colonization of the intestine develops in a successive manner in which bacteria predominate along with lower numbers of archae, yeasts, filamentous fungi, parasites, and viruses [2, 3]. Following initial domination by facultative anaerobes, the gut microbiota becomes gradually inhabited by obligate anaerobes which will remain its major constituents during adult life [4–7]. Triggered by the growing number of 16S ribosomal RNA (rRNA)-based approaches, insights in the evolutionary diversity of the human adult gut flora has changed drastically in recent years. Based on a delineation level of 98% 16S rRNA gene sequence similarity, current estimates indicate that the human gastrointestinal tract encompasses more than 1000 bacterial phylogenetic types, also referred to as phylotypes or “molecular species” [8–10]. These taxonomic inventory studies have revealed that the gut microbiota in adults is largely dominated by members of only two bacterial phyla, that is, the Bacteroidetes and the Firmicutes, and one member of the archaea, *Methanobrevibacter smithii*. Through a complex network of mutualistic interactions, the gut microbiota has a profound impact on the host's health by acting as a barrier against pathogens, contributing to the degradation of food components, stimulating the host immune system, and producing a series of essential vitamins, enzymes, and short-chain fatty acids [11–14].

Until a decade ago, knowledge on the taxonomic composition and metabolic activity of the intestinal tract microbiota was mainly based on the use of culture-dependent techniques. Triggered by the growing awareness that only a fraction of the gut microbiota is culturable under laboratory conditions, various culture-independent methods have been evaluated in intestinal microbial ecology [15–19]. Depending on the scientific rationale and technical design of the study, molecular approaches for assessing diversity and dynamics of intestinal microbiota include population fingerprinting [this review], clone libraries [20–23], dot blot hybridization [24, 25], fluorescent in situ hybridization (FISH) [8, 26–29], real-time PCR [30–33], DNA microarrays [34–36], and metagenomics [9, 37–39].

In contrast to several of the aforementioned techniques that specifically target one or more autochthonous members of intestinal tract or that require analysis of large and complex datasets, population fingerprinting is a universal concept that allows one to characterize and monitor intestinal microbiota without preexisting knowledge of its structure or composition. The most commonly used fingerprinting techniques in the field of intestinal microbiology are based on the sequence-dependent electrophoresis (SDE) principle and include denaturing gradient gel electrophoresis (DGGE), temperature gradient gel electrophoresis (TGGE), and temporal temperature gradient gel electrophoresis (TTGE). In contrast to conventional gel electrophoresis based on fragment size, the SDE principle relies on the sequence-dependent electrophoretic separation of a mixture of equally sized PCR products in a polyacrylamide gel containing a linear gradient of chemical denaturants (DGGE) or a linear temperature gradient (TGGE and TTGE). This way, separation is achieved by the gradually decreasing electrophoretic mobility of partially melted, double-stranded amplicons in the denaturing gradient. PCR fragments equal in length but with different sequences have a different melting behavior and will stop migrating at different positions along the gel, eventually producing a banding pattern or fingerprint. To a lesser extent, also single-strand conformation polymorphism (SSCP) [40] and terminal-restriction fragment length polymorphism (T-RFLP) [41] analysis have been applied in microbial community profiling. Likewise SDE methods, both these methods rely on PCR amplification of specific target sequences followed by electrophoretic separation of amplicons. Whereas this separation is based on sequence-specific melting behavior of amplicons in SDE analysis, the taxonomic resolution of SSCP and T-RFLP is determined by the secondary structure of ssDNA or by the distribution of endonuclease restriction sites, respectively. The principle of SSCP analysis is essentially based on the sequence-dependent differential intramolecular folding of ssDNA which alters the migration speed of the molecules [42]. The ssDNA fragments originating from PCR amplicons are separated using uniform, low temperature, nondenaturing electrophoresis to maintain the secondary structure of the single-stranded fragments. T-RFLP analysis, on the other hand, is based on a size-dependent electrophoretic separation of digested fluorescently end-labeled PCR products. Upon electrophoresis using either gel- or capillary-based systems, only the “terminal” end-labeled restriction fragments are detected. Although less commonly used than DGGE and related techniques, SSCP [43] and, especially, T-RFLP [44–48] have been applied to study the diversity and dynamics of intestinal microbiota. This review will specifically focus on the use of SDE techniques, and DGGE in particular, in the field of intestinal microbiology.

Since their introduction in microbial ecology in the early 1990s [49], SDE fingerprinting techniques have been employed to analyze microbial communities in a wide range of environments including aquatic sites [50–52], soil [53], fermented foods [54, 55], and the human intestinal tract [this review]. The value of SDE-based fingerprinting methods in intestinal microbiology lays in the fact that they allow pattern-based visualization of the predominant bacterial groups including poorly culturable and currently uncultured bacteria that are considered to represent up to 50–90% of the intestinal microbiota.

This review will deal with all different aspects of SDE methodology including its possibilities and limitations in terms of reproducibility, sensitivity, and data analysis. Through discussion of selected studies that have contributed to the field, an overview will be presented of SDE-based research approaches to study human intestinal ecosystems in relation to the microbial ecology of healthy and disease-affected populations. The scope of this review excludes SDE applications dealing with the human upper gastrointestinal tract or with animal intestinal ecosystems.

## 2. METHODOLOGY

### 2.1. Principle

The principle of SDE techniques relies on the electrophoretic separation of PCR amplicons with equal length in a sequence-specific manner in a polyacrylamide matrix containing a defined denaturing gradient of urea and formamide (DGGE) or temperature gradient (TGGE and TTGE). The temperature gradient in TGGE is created along the length of the gel, whereas in TTGE a temporal temperature gradient is gradually formed during the electrophoresis run. The electrophoretic mobility of double-stranded amplicons in a gel matrix with an increasing denaturing gradient is retarded at a given chemical denaturant concentration or temperature that causes (partial) melting of the sequence region with lowest melting temperature (*T*_*m*_). The physical denaturation of the dsDNA fragment is thus largely determined by its nucleotide sequence and %G+C content and proceeds in discrete portions of the fragment or the so-called melting domains. These domains interfere with the helical structure of the DNA molecule and will eventually halt further migration. Amplicons that are different at the sequence level are likely to display a different melting behavior and will, therefore, stop migrating at different positions along the linear gradient of the gel, which upon visualization will result in band profiles representing the sequence diversity of the amplicon mixture.

In practice, SDE-based community profiling comprises four steps: (i) extraction of total community DNA from the sample; (ii) PCR-controlled amplification using specific oligonucleotide primers; (iii) sequence-dependent electrophoretic separation of the amplicons using either DGGE, TGGE or TTGE; and (iv) fingerprint processing and analysis.

### 2.2. Sampling and total DNA extraction

#### 2.2.1. Sample collection and processing

The endogenous microbiota differs along the length of the intestinal tract [56]. In addition to the longitudinal diversity gradient, also a cross-sectional differentiation of the microbial population has been observed in the lumen, the mucosa, and the epithelium surface [56, 57]. Mainly due to sampling difficulties, the taxonomic composition of these microhabitats in the intestinal tract is poorly documented. Because of this spatial distribution, microbiological data obtained from a subsample of the gut cannot always be extrapolated to the global composition of the entire intestinal microbiota. Most often, fecal samples are used to study the intestinal microbiota because they are the most accessible type of specimen that can be collected from this environment. In specific clinical cases, also luminal endoscopy samples, mucus, biopsies, and stoma liquid can be analyzed.

In most studies, immediate processing of samples is not feasible due to the need for transportation and/or (long-term) storage of the specimen. It has been shown that storage of stool samples at room temperature and even at 4°C showed a substantial reduction in bacterial diversity and the degradation of bacterial DNA after 8 hours [58]. Therefore, it is generally recommended that colon samples should be (deep-)frozen immediately upon collection and stored at maximally −20°C and preferably at −70°C, until further processing. However, it should be kept in mind that repeated freezing and thawing of samples can have negative effects on bacterial viability and recovery rates [59–61]. Although poorly documented, the impact of subsequent sample manipulations on DNA extraction and the yield and quality of the resulting DNA probably is less dramatic [62].

#### 2.2.2. Extraction of community DNA

An efficient, reproducible, and high-yield method for total DNA extraction is indispensable in order to obtain a representative view of the actual microbial composition of an intestinal sample. The most crucial step in any DNA extraction procedure is cell lysis. A series of methods including commercial kits and inhouse laboratory protocols have been described and evaluated for the extraction of total bacterial DNA or RNA from intestinal samples making use of chemical, mechanical (e.g., beads), and/or enzymatic lysis [63–67]. Because not all members of the intestinal microbiota display the same sensitivity to the lysis conditions of a given procedure, it is extremely difficult, if not impossible, to extract DNA from all constituting species with the same efficiency. Furthermore, the DNA isolation procedure should also be able to remove potential PCR inhibitors that may be present in fecal samples such as phenols, bile salts, degradation products of hemoglobin, and complex polysaccharides of plant origin. The selection criteria usually applied to evaluate the efficacy of a DNA extraction method include electrophoretic verification of DNA integrity, determination of DNA yield and quality using spectrophotometric analysis and quality control of the obtained SDE profile [64–66]. In addition, the lysis efficiency of different DNA extraction protocols can be compared based on the complexity and band intensity of SDE community fingerprints. Upon extraction, DNA solutions are generally stored at −20°C. The influence of storage conditions and duration of storage on the integrity and quality of total DNA extracts from intestinal samples has not been studied in great detail.

Depending on the type of (clinical) application, high-quality community DNA may need to be obtained from a range of different sample types such as digesta, mucosal, and fecal samples. For this reason, the DNA extraction technique should be carefully selected and possibly further evaluated or optimized with particular attention for the type and number of specimens [66]. In this respect, it may be less appropriate to use commercial DNA extraction kits given the limited possibilities to optimize the procedure, for example, by changing concentrations or composition of extraction reagents. On the other hand, commercial kits can considerably reduce the hands on time compared to more complex inhouse protocols. In Table 1, technical details are given for a number of frequently used total DNA extraction procedures that have been used in SDE-based profiling of human intestinal microbial communities [6, 21, 24, 32, 33, 43, 63–66, 68–109].

### 2.3. Community PCR

Following DNA extraction and purification, multiple primer sets with different taxonomic coverage can be applied for community PCR amplification. The use of universal PCR primers allows any microbial community to be analyzed, although in ecosystems with a high diversity like the intestinal tract only the (pre)dominant constituents will in effect generate a visible band in SDE. In order to focus on a specific subpopulation within the total community, group-specific PCR primers can be used which allow detection of bacterial taxa that are less prevalent in the intestinal tract. Traditionally, universal and specific community PCR primers for SDE applications are designed using the 16S rRNA gene as a target molecule. This preference stems from the fact that the SSU rRNA gene has a mosaic structure composed of both invariant, relatively conserved, and highly variable regions (V regions). In SDE-based population fingerprinting, primers are used that anneal to conserved sequence parts of the gene in order to cover one up to three hypervariable regions. In Table 2, a selection is presented of universal and specific primer sets that have been used in SDE-based profiling of human intestinal microbial communities [6, 30, 64, 65, 69, 71–74, 76, 77, 79–82, 84–86, 88, 90, 91, 94–96, 98, 99, 101, 102, 105–130]. Taking the rumen as model system of complex microbial community, Yu and Morrison [131] systematically compared a set of DGGE profiles obtained with universal primers targeting different V regions. Based on sequence variability and temperature heterogeneity of the lowest *T*_*m*_ domain of the V region and on the number, resolution, and relative intensity of the bands in the resulting DGGE profile, the V3 region was most preferred for analyzing intestinal microbiomes. In addition, the authors recommended to use the V3–V5 or V6–V8 regions if a longer amplicon is preferred. Next to the SSU rRNA gene, also its rRNA counterpart can be coextracted and used as PCR template in SDE analyses of intestinal ecosystems when preceded by reverse transcription [70, 71, 75, 93, 95]. In this way, SDE profiles are generated that represent the (pre)dominant metabolically active bacteria based on the assumption that the cells of these organisms generally have a much higher ribosomal RNA content and rRNA/DNA ratio compared to resting cells.

An additional 40-nucleotide GC rich sequence, the so-called GC-clamp, is usually attached to the 5′ end of one or both of the PCR primers and participates in the PCR reaction. This way, the GC-tail generated at the end of the amplicon will prevent complete denaturation of the product and is necessary to obtain a stable melting behavior of the fragments during electrophoresis [49, 132, 133]. GC-clamps can vary in sequence, length, and location [100, 134–136], and their design needs to be based on the target sequence and the primers used. Mutation analysis data have shown that GC-clamps have the strongest effect on the melting properties of short fragments (<300 bp) and that this effect may be drastically reduced for large fragments (>400 bp) [136]. Also, it has been demonstrated that a GC-clamp length of 60 bp may be efficient for detection of fragments with a *T*_*m*_ value close to 80°C whereas fragments with *T*_*m*_ > 80°C may require longer GC-clamps in combination with naturally occurring high-melting (thus GC-rich) domains [136].

### 2.4. Sequence-dependent electrophoresis

#### 2.4.1. Electrophoresis conditions

Essentially, a DGGE system consists of a heated buffer tank operated under strict control of temperature and stable buffer circulation. Several systems are currently available, of which DCode (Bio-Rad Laboratories; http://www.bio-rad.com/), INGENYphorU (Ingeny; http://www.ingeny.com/), and DGGEK-1001/2001/2401/4001/4801 (CBS Scientific; http://www.cbsscientific.com/) appear to be most commonly used. The apparatus provided by Bio-Rad and Ingeny can also be applied for TTGE analysis whereas for TGGE a temperature gradient block should be integrated in the system. In case a high number of samples need to be analyzed such as in monitoring studies, the sample capacity of the system is an important criterion. The maximum capacity per run for the three aforementioned systems varies from 60 (DCode), 96 (INGENYphorU) to 128 (DGGEK-4801) samples.

In general, DGGE makes use of parallel gel electrophoretic systems that have an increasing vertical gradient of denaturants parallel to the direction of electrophoresis. In many studies, the optimal denaturing gradient yielding the highest resolution is first determined by perpendicular gradient gels. For this purpose, one sample containing one or more PCR fragments is electrophoretically separated across a denaturing gradient perpendicular to the direction of the electric field resulting in sigmoid-shaped curves. From these gels, the intermediate range of denaturant concentration, where different electrophoretic mobilities between PCR products are obtained, is considered the optimal gradient of denaturants for multilane analysis in parallel DGGE. The optimal time of electrophoresis can be determined through a “time travel” experiment during which a mixture of PCR fragments is loaded onto a parallel gel at constant time intervals. The optimal duration of a DGGE run can be derived from the time needed to obtain maximal separation of amplicons.

A detailed procedure to cast and run DGGE gels has been described by Muyzer et al. [134, 137]. Essentially, the desired low and high concentration of denaturing solution is obtained by mixing zero (0%) and high-concentration (80–100%) denaturing acrylamide solutions in appropriate ratios. Upon the addition of ammoniumpersulphate and tetramethylethylenediamine, the mixture is poured between two vertical glass plates in order to generate a linear denaturing gradient. The concentration of acrylamide usually ranges from 6–12% and depends on the size range of the fragments to be separated. In general, the high-concentration denaturing solution contains 7-8 M urea and 20–40% formamide. Electrophoresis is mostly carried out in 0.5× or 1× TAE-buffer at a fixed voltage between 50 V and 250 V and a constant temperature between 55 and 65°C. Run times generally range from 3–17 hours, although longer run times with lower voltages tend to produce better quality gels.

In the case of TGGE and TTGE, a linearly increasing temperature gradient parallel to the electrophoresis direction or formed during the length of electrophoresis, respectively, is applied in combination with a uniform, high-denaturant polyacrylamide gel to separate PCR fragments. To determine the temperature range for parallel TGGE or TTGE analysis, a melting profile of the DNA sequence can be generated using specialized software (e.g., Poland analysis software; http://www.biophys.uni-duesseldorf.de/local/POLAND/poland.html). The optimal temperature gradient is theoretically delineated by the lowest and highest *T*_*m*_ values obtained in the melting profile. The theoretical *T*_*m*_ values can be lowered by adding denaturing components to the gel, for example, one mole of urea will lower the theoretical *T*_*m*_ with 2°C [138, 139]. In general, a 6–8 M urea gel is used in combination with a typical temperature range between 35 and 70°C.

Unlike many other fingerprinting methods that make use of commercially available size standards, SDE techniques suffer from a lack of consensus regarding standards for normalization. Because denaturing gradients can slightly vary between different gels, a standard reference composed of amplicons from pure cultures that spans a maximal range of the applied gradient should be routinely included at several fixed internal positions on every gel to allow data normalization and gel-to-gel comparison with a high degree of confidence. Neufeld and Mohn [140] proposed an approach which facilitated and improved normalization of samples from multiple gels by including standards in each lane instead of using interlane standards. These intralane standards contain fluorescent tags incorporated in the primers that excitate at another wavelength than that of the fluorescent molecules attached to the unknown PCR product. Furthermore, the application of fluorophore-labeled primers does not require gel staining following electrophoresis, which improves the overall sensitivity of the population fingerprinting procedure and enables additional DGGE versatility including simultaneous analysis of DNA- and RNA-derived mixtures in the same lane.

#### 2.4.2. Gel staining

Upon electrophoresis, gels are stained and digitally captured for further analysis. Three staining agents are commonly used to visualize fragments. Originally, SDE gels were stained with ethidium bromide (EtBr) given its widespread use as an intercalating fluorescent dye used to detect nucleic acids. The next generation of fluorescent nucleic acid dyes such as SYBR Green and similar stains offer an increased sensitivity compared to EtBr due to a lower overall background signal allowing detection of DNA fragments at lower concentrations [64, 134, 137]. Additional advantages of these newer dyes are that they are generally considered to be less toxic or mutagenic than EtBr and can be excited by wavelengths above 400 nm which enables the use of non-UV illumination. One specific member of the SYBR Green family, SYBR Gold, binds to both dsDNA and ssDNA. This specific feature may further enhance the detection sensitivity since DNA amplicons in the SDE gels are partially single stranded. Although less commonly applied, silver staining is generally considered the most sensitive staining procedure. Following DNA fixation with ethanol and acid (e.g., nitric acid), Ag^+^ ions in silver nitrate are selectively reduced under alkaline conditions by formaldehyde to metallic silver (Ag) that is visualized as a black precipitate. Potential drawbacks of this procedure include the fact that silver stained gels impede subsequent blotting experiments or band sequence analysis and the aspecific detection of protein components such as BSA and *Taq* polymerase present in the PCR mix which may generate additional background signals [134, 137].

### 2.5. Data analysis

Normalized SDE fingerprints can be analyzed visually and/or numerically. Visual interpretation is attainable when only a limited number of profiles with low complexity are to be compared. However, once banding patterns become more complex such as those obtained from intestinal samples or when the number of profiles increases (e.g., in the course of monitoring studies), analysis of SDE fingerprints requires implementation of numerical methods [141]. For this purpose, digitized SDE gels are further processed using dedicated image analysis software like GelCompar and BioNumerics (Applied Maths; http://www.applied-maths.com/), Quantity One and Molecular Analyst (Bio-Rad Laboratories), GeneTools (Syngene; http://www.syngene.com/), and Photo-Capt (Vilber Lourmat; http://www.vilber.com/). These programs permit numerical analysis of band patterns and usually also include statistical approaches for data interpretation. Programs that have been used specifically for statistical analysis of SDE fingerprint data include R (http://www.r-project.org/) and DGGESTAT (developed at the Netherlands Institute for Ecological Research, NIOO-KNAW, Nieuwersluis, The Netherlands).

#### 2.5.1. Diversity and similarity analysis

Most commonly, numerical analysis of SDE profiles relies on the use of diversity indices and/or cluster analysis. Diversity measures for fingerprint analysis such as the Simpson index and the Shannon-Wiener/Weaver index express the degree of ecosystem diversity as a function of band profile complexity but fail to express similarity between profiles based on band positions. Hierarchic clustering algorithms such as unweighted pair-group method using arithmetic averages (UPGMA) produce a visual representation of the similarity between SDE profiles expressed as similarity indices, for example, using the curve-based Pearson product-moment correlation coefficient, the band-based Dice coefficient, or Sorenson's pairwise coefficient. Other authors have used multivariate ordination methods such as nonmetric multidimensional scaling [142, 143], principal component analysis [109, 144], correspondence analysis [145], canonical variate analysis [146], and canonical correspondence analysis [147]. These methods are used for integration of complex datasets such as the bands in an SDE pattern into new mathematical variables which can be projected into a few-dimension perspective or reduced space. A more detailed description of these statistical procedures has been reported elsewhere [148]. Gafan et al. [149] evaluated the use of logistic regression for statistical analysis of complex DGGE profiles. This analysis method takes into consideration the outcome in addition to differences in overall band profile complexity and individual band positions. It is beyond doubt that the list of numerical approaches and statistical tools for analysis of SDE profiles will further expand in the coming years. Although the choice of method(s) is depending on the aim of the study and on the complexity of the ecosystem, community fingerprints generally include more information than are usually revealed with currently available methods. For this reason, more efforts should be put in the development of new and extended processing methods for complex SDE data.

#### 2.5.2. Identification analysis

Next to the first SDE analysis level based on the use of diversity and similarity coefficients, a second level can be defined that allows one to identify and monitor specific members of the intestinal ecosystem. Essentially, identification of individual bands in SDE fingerprints may be obtained by band position analysis (BPA) and/or through band sequencing analysis. Essentially, BPA relies on the comparison of migration distances of band fragments from taxonomically well-characterized reference strains with those of unknown bands present in the sample profiles. BPA can either be performed by analyzing samples and reference strains in adjacent lanes on the same gel (i.e., comigration analysis) or by comparing unknown band positions with those of reference strains present in a user-generated SDE database. In intestinal ecosystems, BPA-based identification may not always yield a conclusive result given the possibility that a single band may consist of multiple amplicons from different species or that two or more (phylogenetically related) species are characterized by the same band position in the sample profile. Ideally, each band position in a sample profile should represent one species. In practice, however, the multioperon effect observed for some taxa when using 16S rRNA gene primers may lead to an overestimation of the number of predominant species in the sample (e.g., see Section 4.2). In contrast to SDE profiles obtained with universal primers, identification of bands in subpopulation profiles by BPA may be more feasible. Application of SDE using group-specific primers for the genera *Bacteroides* [81, 90] and *Bifidobacterium* [74, 90, 108, 117] showed that species identities can be resolved by means of BPA. Temmerman et al. [117] described a protocol to identify bifidobacterial communities based on a nested-PCR-DGGE approach comprising a *Bifidobacterium*-specific PCR step followed by a second PCR step in which both the V3 and V6–V8 regions of the 16S rRNA gene were amplified. A mix of both amplicons was analyzed on a DGGE gel, after which band positions were compared with a user-generated database of reference strains.

Identification results from BPA can or even should be verified by band sequencing, and may help to determine the phylogenetic affiliation of unknown bands. Various procedures have been described to excise and recover PCR fragments from the polyacrylamide gel matrix ranging from conventional elution in electrophoresis buffer to specialized protocols using diffusion buffers and commercial kits [74]. A critical postextraction step during this process concerns the reamplification and subsequent SDE analysis of the excised fragment together with the original environmental sample in order to verify if the correct band was extracted. Upon confirmation, the recovered PCR fragments can be directly sequenced without additional cloning. Subsequent identification of the obtained sequence information can be achieved by comparison with sequences stored in public databases, for example, EMBL (http://www.ebi.ac.uk/embl/) or GenBank (http://www.ncbi.nlm.nih.gov/genbank).

As further discussed below, the significance of the obtained species information is dependent on the length of the fragment and the hypervariable region it represents in the target gene. This sequence information can also be employed to develop probes for application in FISH and real-time PCR assays to detect and quantify the target organisms. Next to sequencing analysis, identities of individual bands in SDE profiles can also be revealed by Southern hybridization with taxonomic probes [150].

## 3. ANALYSIS OF HUMAN INTESTINAL MICROBIOTA

The human intestinal tract harbors a highly dense and complex microbial community which plays a pivotal role in maintaining the health status of the gut. Despite the fact that SDE-based methods only allow a superficial view on the microbial diversity and population dynamics of what is considered the predominant part of complex ecosystems, their use in the field of intestinal microbiology has increased exponentially over the past 10 years. The following section aims at reviewing the main contributions of SDE population fingerprinting to our current knowledge on the composition and ecological balance of the human intestinal microbiota linked to health, disease, and dietary intervention.

### 3.1. Normal intestinal microbiota

Next to a relative minority of organisms belonging to other microbial domains, the human intestinal microbiota mainly consists of bacteria. Although the major site of microbial fermentation is the large intestine (colon), bacterial populations are encountered along the total length of the digestive tract. Starting from the upper bowel, bacterial concentrations gradually increase up to 10^11^-10^12^/g in the colon. Parallel to the increase in bacterial density, also the bacterial diversity expands from the small intestine to the colon [151, 152]. From the community point of view, it is important to realize that the intestinal ecosystem evolves from an initially sterile system that becomes successively colonized by various microorganisms.

#### 3.1.1. From newborn to adult

Several studies have used SDE-based techniques to monitor the development of the newborn gut microbiota in humans [6, 96, 101, 102, 106, 107]. At birth, the initially sterile gut becomes inhabited by a variety of bacterial taxa. Succession continues during weaning until a more complex and stable microbiota is established. Two studies by Favier et al. [6, 96] have shown that the intestinal bacterial community of newborns is extremely unstable as evidenced by the fact that many dominant bands in DGGE profiles of fecal samples from healthy full-term babies reduced in intensity, gradually disappeared after a few days and were substituted by other bands. In the first weeks of life, DGGE profiles obtained with universal 16S rRNA gene V6–V8 primers consisted of only a few bands but progressively increased in complexity over time. In combination with clone libraries constructed from 16S rRNA gene sequences, identification of bacterial species corresponding to specific bands in DGGE profiles was possible by BPA. This approach indicated that *E. coli* and *Clostridium* spp. were the main groups among the initial colonizers, which were rapidly replaced by a more complex microbiota consisting of *Bifidobacterium, Clostridium, Enterococcus, Ruminococcus, Enterobacter, Streptococcus, Bacteroides*, and *Actinomyces*. The diversity revealed by DGGE analysis was fairly consistent with previous insights in infant succession patterns based on traditional culture studies [5, 7]. In addition, the successive colonization of the infant gut by bifidobacteria was monitored during the first five months after birth using *Bifidobacterium*-specific primers [96]. Whereas some subjects showed very stable DGGE profiles, others revealed temporal variation in their bifidobacterial population. At each point in time, one to four *Bifidobacterium*-related DGGE bands were observed which always included *Bifidobacterium infantis*. In another study, the dynamics of the developing bacterial community in the neonatal intestinal tract of nine Japanese infants was monitored during the first two months of life [102]. Although the development of individual species was different among the subjects, DGGE profiles of the predominant fecal microbiota together with 16S rRNA gene clone library sequencing revealed a global stepwise evolution from an aerobic to an anaerobic microbial ecosystem. The aerobic organisms that were initially present such as *Pseudomonas* were immediately replaced by facultative anaerobes including *Enterococcus, Streptococcus*, and *Enterobacteriaceae* during the first month. Finally, strictly anaerobic bifidobacteria and clostridia appeared. The establishment and succession of bacterial communities in hospitalized preterm infants tend to follow a different pattern compared to full-term infants [101]. Fecal samples from 29 preterm infants hospitalized in a neonatal intensive care unit and 15 full-term infants were analyzed using DGGE to characterize and compare bacterial succession of the dominant bacterial species in the large intestine. In the first four weeks of life, DGGE patterns increased in complexity over time for all preterm infants. During this observation period, the intraindividual band pattern similarity increased over time as indicated by an increase in Sorenson's pairwise similarity coefficient (*C*_*s*_) from 0 to 80%. In addition, also the interindividual *C*_*s*_ values increased (18.1 to 57.4%) all of which indicated the acquisition of a highly similar bacterial community in these infants. In contrast, breastfed full-term infants showed a considerably lower interindividual *C*_*s*_ value (11.2%). The strikingly high similarity between bacterial communities from different preterm infants was considered to be associated with hospitalization because the major bacterial groups identified by DGGE BPA belonged to taxa that are routinely isolated in baby care units such as *E. coli, Enterococcus* spp., and *Klebsiella pneumoniae*. This finding thus indicates that the initial colonization of the newborn's intestinal tract is highly dependent on the immediate environment of the individual. In another study assessing the global diversity of the fecal microbiota of preterm infants (*n* = 16), a remarkably low-species diversity and high-interindividual variability were reported [106]. The low-bacterial diversity was revealed by random sequencing of 16S rRNA gene clones and TTGE analysis. The main fecal groups encountered here included members of the *Enterobacteriaceae* family and of the genera *Enterococcus, Streptococcus*, and *Staphylococcus*. Seven out of 16 preterm infants were colonized by anaerobes, of which four infants were shown to harbor bifidobacteria.

Several studies have documented that bifidobacteria predominate in the fecal flora of breastfed babies, whereas in formulafed infants, other bacterial groups such as coliforms, enterococci, and *Bacteroides* represent the main constituents [7, 153]. In contrast, the possible effect of dietary supplementation in the intestinal development of nursing infants is less well understood. In a longitudinal study, TTGE was used to monitor the predominant and bifidobacterial microbiota of 11 Algerian infants during breastfeeding, breastfeeding with artificial milk supplementation (weaning) and artificial milk alone (postweaning, i.e., cessation of breastfeeding) [107]. In the TTGE profiles, the major bands were assigned by subsequent cloning and sequencing to *E. coli, Ruminococcus* spp., and several *Bifidobacterium* species including *B. longum, B. infantis*, and *B. breve*. Both for the bacterial and bifidobacterial TTGE profiles, distance analysis indicated the expected maturation of the faecal microbiota between 5 and 20 weeks of age, but did not reveal any correlation with the dietary supplementation. Despite a high-interindividual variability, it was observed that the composition of the faecal microbiota appeared more homogenous after weaning which may suggest a correlation with the cessation of breastfeeding. In another study, 65 10-month old infants were included in a randomized dietary intervention study that compared the effect of cow's milk (CM) with infant formula (IF) with or without fish oil (FO) supplement on the diversity of the fecal microbiota [80]. Based on clustering analysis of V3- and V6–V8-16S rDNA DGGE profiling using the Pearson correlation coefficient, it was reported that supplementation of CM or IF appeared to have an influence on the composition of the intestinal microbiota whereas FO intake only showed an effect in the CM group. The authors speculated that these differences may be influenced by the intake of iron and *n*-3 polyunsaturated fatty acids, respectively, but further indepth analysis of the DGGE profiles in combination with other molecular tools is required to substantiate this hypothesis.

Besides the influence of environmental and dietary factors, also the host genotype may have a significant effect on the species composition of the intestinal microbiota. Stewart et al. [84] used TTGE analysis of the predominant bacterial biota to investigate the influence of host genotype on the fecal microbiota in genetically related and unrelated children. In that study, TTGE profiles of identical twin pairs (*n* = 13), fraternal twin pairs (*n* = 7), and unrelated control pairs (*n* = 12) were compared both visually and numerically. Although the community fingerprints of each individual were unique, increased levels of similarity were found between TTGE profiles of genetically related individuals, with the highest similarity values obtained for genetically identical twins (median *C*_*s*_ of 82%) which was significantly different from fraternal twin pairs (median *C*_*s*_ of 68%) and from the unrelated control group (median *C*_*s*_ of 45%). The results of this TTGE study thus suggested that host genetics can have an impact on the composition of the predominant fecal bacterial community in children. Likewise, DGGE analysis of the dominant intestinal microbiota amongst adults displaying varying degrees of genetic relatedness showed that the host genotype had a significant effect on the species composition of the intestinal community [122].

Upon succession, it is thought that a relatively stable intestinal community is established in the adult intestine that appears to be specific for each individual. Zoetendal et al. [95] were the first to report on the stability and uniqueness of the predominant human adult fecal microbiota that can be visualized with SDE-based approaches. TGGE analysis of fecal samples from two healthy individuals showed stable profiles over a period of at least six months which in addition were unique for each individual. These findings were consolidated in a later study [64] in which the host specificity and temporal stability of the DGGE patterns was demonstrated for four subjects over a 16-week period by visual inspection and clustering analysis. In the latter study, also the temporal stability of selected subpopulations was monitored using group-specific primers. DGGE profiles obtained with primers designed to visualize the *Lactobacillus-Leuconostoc-Pediococcus-Weissella*-group tended to show strong temporal variations. Among other autochthonous groups such as the *Bacteroides* *fragilis* subgroup, however, DGGE profiling using group-specific primers did not reveal such variations. Importantly, the specificity of these group-specific primers was only validated using a set of taxonomic reference strains. A more elaborated strategy was followed in the validation of DNA- and RNA-based DGGE protocols specifically designed to assess the diversity and stability of the *Clostridium coccoides-Eubacterium rectale* (clostridial phylogenetic cluster XIVa) group in fecal samples [70]. In that study, the specificity of the Ccoc-f and Ccoc-r primers was assessed by constructing a clone library in which all 205 DGGE fragments proved to belong to the *Clostridium* cluster XIVa. The authors concluded that the members of this cluster, representing one of the most dominant bacterial groups in the normal intestinal microbiota, followed the same pattern of relative stability as the total predominant population in 12 healthy Finnish adults during six months to two years. Although using protocols differing in sample type, SDE method and primer target, the current view on the uniqueness and temporal stability of the predominant intestinal flora in adult individuals has also been confirmed in other human volunteer studies using SDE-based analyses of fecal samples [77, 81, 83, 98, 99, 118, 154] and mucosa samples originating from different parts of the large intestine [79, 98, 123].

Although the vast majority of SDE-based studies in intestinal microbiology rely on direct DNA extraction from human samples in order to obtain a culture-independent inventory of the microbial diversity, there has also been interest in using DGGE and related fingerprinting techniques to specifically explore the composition of culturable intestinal subpopulations. For instance, DGGE analyses of resuspended bacterial biomass obtained from agar plates of different media selective and nonselective for lactic acid bacteria (LAB) have been used to evaluate the choice of medium and incubation conditions on LAB recovery and to gain insight in the diversity of culturable fecal LAB in healthy adults [129].

#### 3.1.2. Spatial distribution

The different physicochemical conditions such as pH and concentration of fermentation products prevailing in the ascending, transverse, and descending parts of the colon [155] suggest that also the bacterial composition in each of these three compartments is unique. However, this assumption is not substantiated by SDE-based studies [30, 79, 91, 115, 123]. In most of these studies, DGGE and TTGE fingerprint profiles reflecting the predominant bacterial communities in biopsy samples from different sites of the colon were host specific but highly similar between sites. These findings may indicate that the spatial distribution of at least the predominant mucosa-associated bacterial community is relatively uniform along the length of the colon and its physicochemical gradient. Nielsen et al. [79] reported that DGGE profiles of the bifidobacterial community were relatively simple and consisted of one or two bands for most of the sites sampled along the length of the colon. However, the mucosa-associated subcommunity encompassing the genera *Lactobacillus, Leuconostoc, Weissella, Pediococcus*, and *Aerococcus* produced relatively complex DGGE profiles that varied between hosts and between sampled sites in the colon. In contrast, Zoetendal et al. [123] obtained DGGE profiles with low diversity and little or no variation along the colon when using the same set of group-specific PCR primers. Presumably, the contradictory findings of the two aforementioned studies are due to differences in sampling procedure, DNA extraction method, and/or composition of the subject group.

Given the fact that each individual displays a unique fecal SDE fingerprint [64, 95], investigation into spatial distribution should preferably be based on analysis of a series of site-specific biopsy samples from the same individual. To some extent, this may explain why interindividual comparison of DGGE profiles of single biopsy samples from different sites did not provide any evidence for the existence of site-specific colonization patterns in the human colon [30].

A number of studies have also investigated to what extent the composition of the fecal microbiota reflects the composition of the mucosa-associated colonic microbiota [91, 123]. In these studies, the DGGE/TTGE profiles of amplicons of the variable V6–V8 region of the 16S rRNA gene reflecting the predominant bacterial community of biopsy samples differed significantly from those of fecal samples within the same individual, suggesting that different bacterial populations are dominating the human mucosa and feces. The population diversity revealed by SDE-based community fingerprinting of fecal samples may thus not necessarily reflect the ecosystem composition in other parts of the intestinal tract including the colonic mucosa. This leads to the conclusion that the most accurate information on the diversity and stability of local intestinal communities can thus only be obtained by taking samples through endoscopy or during colonic surgery.

### 3.2. Intestinal disorders

The pathogenesis of many chronic intestinal disorders and even a number of nonintestinal diseases is believed to be directly or indirectly linked to some members of the indigenous microbiota. Several studies have implemented an SDE-based approach to analyze and monitor the composition and temporal stability of the intestinal microbiota of patients suffering from gut disorders. As an initial approach, SDE techniques permit a rapid and global assessment of microbial diversity without previous knowledge of the composition and are well suited to analyze intestinal microbiota in relation to different experimental conditions and parameters such as healthy versus disease status, active versus quiescent disease phase, different segments of the intestinal tract and response to nutritional or therapeutic interventions. Moreover, the combined use of SDE techniques and quantitative assays such as real-time PCR and FISH that allow to determine the relative concentration of specific indicator organisms offers great potential in this type of studies. The following sections of this review are based on a selected number of studies that have implemented SDE-based methods to assess the potential role of the intestinal microbiota in the (etio)pathogenesis of chronic intestinal disorders.

#### 3.2.1. Inflammatory bowel disease

Although the exact etiology of inflammatory bowel disease (IBD) is not known to date, it is generally assumed to result from an inappropriate response of the mucosal immune system to the normal enteric microbiota in a genetically susceptible individual [156]. It has been hypothesized that specific genetic polymorphisms, such as those in intracellular NOD2 sensors with abnormal function, results in a failure to efficiently regulate expression of Paneth cell-derived antimicrobial peptides [157, 158]. The partial loss of this protective function may allow commensals to damage epithelial cells hereby inducing an inflammatory response. Crohn's disease (CD) and ulcerative colitis (UC) are the two major IBD phenotypes and are characterized by chronic inflammation of the intestinal tract lining which causes severe watery and bloody diarrhoea and abdominal pain [156]. Whereas CD can virtually affect any segment of the intestinal tract, UC is usually confined to the colon and rectum.

The majority of SDE-based studies on IBD have primarily attempted to find differences between CD/UC and healthy fecal or mucosal populations. As such, V3–V5-16S rDNA DGGE profiling and subsequent band sequencing analysis of fresh mucosal biopsy samples revealed a significantly higher prevalence of *Clostridium* spp., *Ruminococcus torques*, and *E. coli* in samples from CD patients (*n* = 19) compared to healthy specimens (*n* = 15) [159]. In turn, the butyrate-producing *Faecalibacterium prausnitzii* was more frequently encountered in the latter group. Overall, DGGE fingerprints of mucosal CD populations displayed a higher patient-to-patient variability compared to healthy subjects. The authors postulated that this difference may reflect the difficulty of patients genetically predisposed to CD to maintain and regulate a stable intestinal microbiota. A study of Bibiloni et al. [160] showed that the phylogenetic composition of biopsy-associated bacteria differed between newly diagnosed untreated CD (*n* = 20) and UC patients (*n* = 15) and healthy subjects (*n* = 14). Biopsies collected from inflamed and noninflamed sites of the terminal ileum and various colonic regions were analyzed by DGGE, 16S rRNA gene clone libraries, and qualitative and quantitative PCR for detection of selected bacterial groups. DGGE profiles of universal V3-16S rRNA gene amplicons were very similar within each subject (mean 85.0 ± 2.4%), irrespective of the intestinal region. However, enumeration by quantitative PCR revealed approximately double numbers of biopsy-associated bacteria for UC patients than CD patients and healthy subjects. In addition, the clone library composition indicated that the composition of biopsy populations in UC and CD patients (*P* < .05), and those from healthy subjects (*P* = .05) were statistically different. This comparison highlighted a significantly higher prevalence of unclassified members of the phylum Bacteroidetes in CD patients, which may indicate that UC and CD are bacteriologically distinct diseases.

Depending on the individual effectiveness, IBD patients undergoing immunomodulatory therapy continuously balance between active disease and remission status. However, it is unclear if and in what way the intestinal microbiota of these patients undergoes compositional changes during these subsequent transitions. In this context, Seksik et al. [24] monitored the fecal microbiota of patients with active colonic CD (*n* = 8), patients in remission (*n* = 9), and healthy volunteers (*n* = 16). TTGE profiles of universal 16S rRNA gene V6–V8 amplicons were very stable over time in the healthy controls but varied markedly for a number of patients (*n* = 4) who were monitored during both active and quiescent phase of CD. Fecal TTGE profiles of these four patients revealed only a slight decrease in the number of bands during the active phase (mean loss of 1.7 ± 2.7 bands), which indicated that the predominant fecal microbiota retained a high degree of diversity in both phases. Based on TTGE band profile composition, no specific bacterial groups could be assigned to active or quiescent CD state. In contrast, quantitative dot blot hybridization of stool samples showed that the fecal microbiota in patients with CD (both active and inactive) differed considerably from those of healthy subjects. Both the *Bacteroides* group (including the genera *Bacteroides, Prevotella*, and *Porphyromonas*) and the bifidobacteria tended to be less represented in CD patients whereas significantly more enterobacteria could be detected. In addition, approximately 30% of the endogenous microbiota of CD patients did not belong to the dominant phylogenetic groups commonly found in healthy controls.

Although currently available data from SDE profiling and other molecular tools implicate a role of intestinal bacteria in CD pathogenesis, a detrimental effect of localized qualitative dysbiosis in CD-associated ulceration has so far not be demonstrated by community fingerprinting. TTGE analysis of biopsy samples of ulcerated and adjacent nonulcerated mucosa of 15 patients with active CD did not reveal qualitative differences in the dominant bacterial population profiles (V6–V8 region of the 16S rRNA gene) within a given patient although a high biodiversity was retained in both cases [92]. Mean similarity values between TTGE profiles of ulcerated and nonulcerated mucosa expressed with the Pearson correlation coefficient did not differ significantly across the different intestinal segments (ileum, right colon, left colon, and rectum) analyzed and ranged from 95.2 ± 4.2% to 97.9 ± 1.7%. Solely based on TTGE analysis using universal primers, it thus appears that local ulceration is not associated with pronounced variation in local bacterial diversity. This conclusion was further substantiated in a later study by the same group on the basis of V3-V4-16S rDNA TTGE profiling and FISH analysis [94]. Also in other studies applying SDE-based population fingerprinting, no particular mucosa-associated microbial pattern could be linked to the (etio)pathogenesis of IBD [91, 123]. Possibly, local dysbiosis among less predominant species may play a role in the pathogenesis of ulceration. Because these minor differences in diversity will largely remain undetected in SDE fingerprinting using universal PCR primers or are difficult to reveal by routine methods for band pattern analysis, future studies should employ group-specific primers to focus on the composition of specific subpopulations and/or should use more indepth mathematical approaches for differential profile analysis.

Whereas most studies concentrated on the inventorization and monitoring of bacterial groups potentially associated with CD, very few studies aimed to address the same question within the metabolically active compartment of the gut microbiota. Sokol et al. [93] analyzed the biodiversity of active bacteria in the dominant fecal microbiota of UC patients (*n* = 9) in comparison with that of healthy subjects (*n* = 9) by applying DNA- and RNA-based TTGE analysis of V6–V8 ribosomal amplicons. The number of bands in DNA-derived TGGE profiles were significantly higher than in RNA-derived profiles for UC patients (15.3 ± 3.2 and 9.1 ± 2.8 bands, resp.) but not for controls (18.3 ± 5.0 and 14.7 ± 5.1 bands, resp.) which indicated a reduction in the biodiversity of the active portion of the fecal microbiota in UC patients relative to healthy controls. Irrespective of the initial template (RNA or DNA), Pearson-UPGMA clustering analysis of TGGE profiles tended to group the samples on the basis of their clinical affiliation (UC versus controls) suggesting that each group has its specific bacterial signature. Interindividual comparison of the “active” microbiota (RNA-derived profiles) revealed a band that was significantly associated with UC patients (89% versus 22% for controls). Sequence analysis attributed this band to *E. coli* or related enterobacteria. Clearly, the possible pathophysiological role of this overrepresentation in the active microbiota of UC patients should be further assessed during remission and within the mucosa-associated microbiota.

#### 3.2.2. Other intestinal disorders

Irritable bowel syndrome (IBS) is an intestinal disorder that is characterized by bowel dysfunction and pain [161, 162]. IBS is a very heterogeneous condition and includes three symptom categories: (i) diarrhoea-dominant, (ii) constipation-dominant, and (iii) alternating type [163, 164]. Although the pathophysiology of IBS is not fully understood, it is highly probable that alterations in the diversity and stability of intestinal microbiota play a role in the development and/or maintenance of this disorder [165]. In a Finnish study [121], culture-based techniques and DGGE analysis were employed to compare the composition and temporal stability of the fecal microbiota of 21 IBS patients and 17 healthy controls. Culturing revealed slightly higher coliform numbers as well as an increased aerobe/anaerobe ratio in the IBS group. DGGE analysis of 16S rRNA gene V6–V8 amplicons revealed considerable biodiversity and subject specificity of the predominant microbiota in both study groups, but did not identify IBS-specific bacterial groups. Visual comparison of DGGE fingerprints revealed a higher frequency of temporal instability in the predominant bacterial population of IBS subjects (43%) compared to controls (29%). However, profile similarity analysis using the Pearson correlation coefficient revealed comparable interindividual similarity percentages for both groups with a mean similarity of 87.5 ± 11.2% for the IBS group and 85.7 ± 12.7% for the control group. Still, the instability in some of the IBS subjects could partly be explained by disturbances of the intestinal microbiota due to antibiotic therapy during the study. Moreover, the authors suggested that these findings could be associated with a subset of IBS subjects sharing specific symptoms and thus not necessarily reflect the general microbial status of all IBS patients. In this regard, future studies should include subject groups with well-defined symptom-based IBS parameters to evaluate the association of intestinal instability with specific IBS symptoms or with specific bacterial groups and species. In a subsequent study of the same group [71], the predominant and clostridial fecal microbiota of IBS patients and healthy controls were compared to reveal possible differences in the composition, abundance, and stability of selected groups by applying DNA- and RNA-based DGGE analyses and transcript analysis with the aid of affinity capture, a multiplexed and quantitative hybridization-based technique. Clostridia, that is, *C. histolyticum, C. coccoides-Eubacterium rectale, C. lituseburense*, and *C. leptum*, were shown to represent the dominant fecal microbiota in 26 of the 32 subjects under study, contributing altogether 29–87%. The proportion of the *C. coccoides-E. rectale* group was found to be significantly lower in the constipation-type IBS subjects compared to the controls. Although DNA- and RNA-derived predominant community profiles showed considerable biodiversity and subject-specificity, RNA-based DGGE profiles contained significantly fewer amplicons (16 ± 5 compared to 22 ± 5 amplicons). In addition, only RNA-based DGGE profiles of the IBS subjects indicated higher instability of the bacterial population compared to the control subjects. Although intraindividual temporal instability of the predominant microbiota was observed in both IBS and control subjects (with both DNA- and RNA-based DGGE), only RNA-derived DGGE profiles of IBS subjects showed a broader range in similarity values (39–95%) compared to control subjects (68–94%). When considering symptomatic IBS subgroups, the largest intraindividual variability in DGGE similarity values was observed in the diarrhoea-type subgroup. These observations suggest that clostridial microbiota, in addition to the instability of the active predominant fecal bacterial population (RNA-derived profiles), may be involved in IBS. For future research, the use of group-specific primers in SDE analysis focussing on apparently affected groups (e.g., coliforms and clostridia) could be a valuable and effective approach to identify potential IBS indicator organisms.

The use of SDE-based methodologies to determine the diversity and stability of microbiota in inflammatory diseases has meanwhile expanded from IBD and IBS to other intestinal diseases in which dysbiosis of the human microbiome is thought to play a role such as neonatal necrotizing enterocolitis [116] and coeliac disease [130] or diseases beyond the intestinal system such as (atopic) allergies [82, 85, 166] and ankylosing spondylitis [113].

### 3.3. Intervention studies

Apart from components naturally occurring in a normal diet, also functional foods (including pre- and probiotics) and antimicrobial agents are able to induce beneficial or detrimental changes in intestinal ecosystems. Starting from the first weeks upon birth, the human diet is able to modulate the composition and balance of the intestinal microbiota [7, 153]. The SDE approach is routinely applied in administration studies to monitor the effects on the intestinal microbiota upon consumption of various active components.

#### 3.3.1. Functional foods

The fact that diet is a major factor controlling the human intestinal balance has triggered the development of a new generation of foods specifically designed to strengthen the gut microbiota via modulation. Functional foods include foods and food products with a clearly identifiable health benefit in addition to their basic nutritional value [167]. In functional foods, the addition or incorporation of pro- and/or prebiotic components as active ingredients plays a key role in functional applications aiming at modulation of intestinal microbiota. According to the FAO/WHO definition [168], probiotics are live microorganisms which, when administered in adequate amounts, confer a health benefit for the host. An extended version of this definition is still under debate, including the question whether the live status is truly required for probiotic action [169, 170]. Beneficial effects induced by probiotic activities are mediated either through modulation of the indigenous microbiota or through the immunomodulatory potential of the probiotic strains used. Bacterial cultures incorporated in probiotic products for human consumption commonly—but not exclusively—originate from the intestinal system of healthy (human) subjects and most frequently belong to the bifidobacteria and to LAB such as *Lactobacillus* spp. A prebiotic, on the other hand, is a nondigestible selectively fermented compound that induces specific changes both in the composition and/or the activity of the gastrointestinal microbiota thereby conferring benefits upon host well-being and health [171]. Essentially, the functionality of a prebiotic compound is determined by its potential to stimulate beneficial bacteria indigenous to the gut ecosystem. Complex oligosaccharides are most commonly used as prebiotics including lactulose, galactooligosaccharides (GOS) and fructooligosaccharides (FOS; e.g., oligofructose and inulin). A wide range of beneficial effects have been attributed to probiotics, prebiotics, or a combination thereof (i.e., synbiotics), including modulation of the gut immune system, resistance to microbial infections, antimutagenic/anticarcinogenic effects, reduction of blood ammonia and cholesterol levels, prevention and/or alleviation of diarrhoea and constipation, prevention and reducing symptoms of intestinal chronic disorders, relief of lactose intolerance and increased mineral absorption as reviewed in [172–178].

SDE-based methods have played a key role in human dietary intervention studies aiming at demonstrating the efficacy of functional food components and to substantiate potential health claim. A selection of relevant studies that have contributed to this field is listed in Table 3 [68, 69, 72, 73, 75, 76, 88, 90, 98, 100, 105, 110, 111, 120, 127, 179, 180]. Solely based on findings from SDE analysis, it appears that prebiotic administration can potentially affect the predominant bacterial population of healthy human subjects, whereas most probiotic interventions only seem to induce marked effects in patient groups. This could indicate that some probiotic components may have more of a therapeutic effect in subjects with a disturbed intestinal balance but less effective as general health promoting agents. On the other hand, it should be kept in mind that SDE-based approaches focus on diversity and dynamics of predominant intestinal microbiota, and are as such unsuitable to monitor probiotic interventions that are based on the immunomodulatory potential of the administered organism(s). Bibiloni et al. [110] used DGGE to evaluate the safety and efficacy of the mixed probiotic preparation VSL#3^®^ (http://www.vsl3.com/) consisting of three *Bifidobacterium* strains and five LAB strains (i.e., four *Lactobacillus* strains and one *Streptococcus thermophilus* strain) in patients with active mild to moderate UC. DGGE analysis of V3-16S rRNA gene amplicons generated from biopsies collected from seven patients before and after 6-week VSL#3 administration revealed considerable variation of the predominant microbiota in four out of five patients in remission (mean dice similarity coefficient (*D*_*s*_) of 69.9 ± 12.7%). In contrast, the DGGE profiles of the two patients with continued active disease remained relatively stable after VSL#3 consumption (mean *D*_*s*_ of 92.3 ± 4.1%). Importantly, it should be noted that the study did not report on the temporal stability of biopsy profiles in the absence of probiotic treatment. In another study, the effect of a 4-week administration of the candidate prebiotic di-D-fructofuranose-1, 2′ : 2, 3′-dianhydride (DFA III) on human fecal microbiota was studied by DGGE analysis using universal V3-16S rRNA primers and *Bacteroides fragilis* subgroup-specific primers [73]. Visual and numerical analysis of the DGGE profiles generated with both primer sets revealed no pronounced changes related to DFA III administration in healthy subjects. In a followup long-term human feeding trial (2 to 12 months) with DFA III, however, DGGE profiles of the predominant bacterial population revealed a marked increase in the intensity of bands related to *Bacteroides* spp. [72]. In a study on the effect of 3-week consumption of a GOS-containing probiotic yogurt on the diversity and temporal stability of fecal microbiota in elderly [69], DGGE revealed that the predominant bacterial population and the *Clostridium coccoides-Eubacterium rectale* group remained relatively stable during the study period. In contrast, the *Lactobacillus* group showed temporal variation which confirms previous observations under basal conditions [64].

In the course of probiotic intervention studies, DGGE and related fingerprinting techniques have been used to verify if the administered strain(s) is (are) detectable in intestinal samples [76, 88, 98, 100, 105, 110, 125, 179]. Based on a combination of culture-based methods and 16S rDNA DGGE, Wall et al. [86] even reported the recovery of probiotic strains *Lactobacillus paracasei* NFBC 338 and *B. animalis* subsp. *lactis* Bb12 in ileostomy effluents of two infants without a history of probiotic intake. In this context, it should be noted that SDE fingerprinting is not the most optimal tool for detection of administered strains because of the relatively poor detection limit (especially when using universal primers) and the lack of resolution to discriminate the introduced strain(s) from other strains of the same or highly related autochthonous member species of the intestinal microbiota. More suitable approaches are those applying strain-specific primers (e.g., conventional or real-time PCR) or probes (e.g., fluorescent FISH probes) which will not only provide a higher sensitivity but may also allow relative quantification of the probiotic target [181–184]. On the other hand, it should be kept in mind that all aforementioned DNA-based approaches do not allow to discriminate between living and dead cells and thus do not provide information on probiotic survival throughout the gastrointestinal tract.

In recent years, SDE-based community fingerprinting has been integrated in larger polyphasic studies in combination with conventional culture methods and/or with other molecular culture-independent methods to detect and monitor changes in human intestinal ecosystems upon administration of probiotic, prebiotic, or other (in)organic compounds with claimed functionalities. As such, DGGE and FISH approaches were combined with selective culture methods to evaluate the impact of a 3-week diet supplementation with prebiotic GOS or FOS on the composition and activities of the fecal microbiota of 15 healthy human volunteers [75]. V3-16S rRNA gene DGGE profiles remained relatively stable during the study, whereas clear alterations in response to dietary supplementation were observed in rRNA-DGGE profiles as evidenced by the detection of additional fragments or increased staining intensity of band fragments attributed to *Bifidobacterium adolescentis* and/or *Collinsella aerofaciens*. In contrast, DGGE analysis using genus-specific primers derived from the transaldolase gene generated relatively stable profiles for fecal bifidobacteria. Although the taxonomic composition of the bifidobacterial population was not substantially different and both DGGE and FISH revealed that the *Bifidobacterium* and *Collinsella* populations remained relatively unchanged, rRNA-DGGE provided evidence of increased metabolic activity in response to prebiotic consumption. A combination of DGGE and FISH was also used to investigate the effect of black tea drinking on the fecal microbiota of healthy volunteers with hypercholesterolemy [120]. DGGE of 16S rRNA gene V6–V8 amplicons showed that each subject harboured a specific predominant bacterial population that exhibits little change over time and that was not significantly changed by drinking black tea. Even though black tea did not affect the specific bacterial groups analyzed by FISH (i.e., *Bifidobacterium, Bacteroides* and *Prevotella, Clostridium* phylogenetic clustersIV and XIVa, *Atopobium* group, *Faecalibacterium*-like species and *E. coli*), it did decrease the total amount of bacteria detected by the universal bacterial probe. In a study that combined the use of TGGE and FISH analysis, it was demonstrated that isoflavone supplementation with and without pro- or prebiotics induced significant dynamic changes on the composition of the dominant intestinal microbiota of 39 postmenopausal women [105]. Results of FISH analysis indicated that several of the dominant fecal groups were stimulated by isoflavones alone, whereas TGGE profiling of 16S rRNA gene V6–V8 amplicons revealed marked changes in the predominant intestinal microbiota. Intraindividual comparison of TGGE fingerprints showed a mean Pearson similarity value of 73% before and after one month of isoflavone supplementation. In combination with a pro- or prebiotic compound, isoflavones triggered comparable population changes as evidenced by mean fingerprint similarity values of 71 ± 18% and 68 ± 16% obtained for the probiotic (*Bifidobacterium animalis* DN-173 010) and the prebiotic (FOS) test groups, respectively. In addition, FISH results showed a bifidobacterial increase following prebiotic supplementation, often referred to as the bifidogenic effect. Amongst others [76, 125, 180], the aforementioned studies have demonstrated the potential of using SDE fingerprinting and FISH analyses in a complementary approach to characterize basic interactions between intestinal microbiota and functional food compounds and to quantify subpopulations responding to the introduced component(s).

Next to FISH, also real-time PCR has been used in combination with DGGE to verify and substantiate compositional changes in a semiquantitative manner. The latter two methods were used in an integrated approach to monitor and quantify pronounced changes in fecal microbiota of healthy subjects upon long-term administration of a prebiotic (lactulose), a probiotic (*Saccharomyces boulardii*), and their synbiotic combination [90]. Although the DGGE profiles obtained with the universal V3-16S rRNA gene primers as well as those generated using group-specific primers targeting the *Bacteroides fragilis* subgroup, the genus *Bifidobacterium* and the *Clostridium lituseburense* and *Clostridium coccoides-Eubacterium rectale* groups remained fairly stable, one pronounced change was observed in the universal fingerprint profiles after lactulose ingestion. The DGGE band appearing or intensifying in 27 of the 30 subjects could be assigned to *Bifidobacterium adolescentis* by band position analysis and band sequencing. In subsequent real-time PCR analysis, this finding was correlated to a statistically significant stimulation of total bifidobacteria and of *B. adolescentis*. In contrast, the probiotic yeast *S. boulardii* did not display any detectable universal changes in the DGGE profiles nor influenced bifidobacterial levels. In a double-blind crossover study on the qualitative and quantitative effects of fresh and heat-treated yogurt on the bacterial intestinal microbiota from healthy subjects [68], DGGE profiling revealed overall stability of the predominant bacterial population and the LAB population at baseline, after fresh yogurt intake and after heat-treated yogurt intake. However, real-time PCR with group-specific primers indicated a significantly higher density of LAB and *Clostridium perfringens* and a significant decrease in the density of *Bacteroides* after consumption of both types of yogurt.

#### 3.3.2. Antimicrobial agents

Apart from their generally well-documented therapeutic effects on the site of infection, antimicrobials can also exert a detrimental effect on the microbial balance of the gut ecosystem. So far, studies analyzing the effect of antibiotic therapy on the selection and transmission of antibiotic resistance among pathogens and commensals within the human intestinal microbiota have mainly relied on culture-dependent approaches [185–187] which are highly restricted by the selectivity of the media used. In this respect, SDE-based techniques provide a more suitable approach to monitor the effects of antimicrobial agents on the total community structure of intestinal microbiota.

In several human studies mostly focussing on infant populations, DGGE analysis has revealed drastic alterations among the indigenous bacterial diversity upon therapy with various antimicrobials [77, 96, 101, 102]. Antimicrobial-induced disruptions of fingerprinting profiles were generally accompanied by a reduction in band numbers suggesting an overall decrease in predominant intestinal ecosystem diversity. Favier et al. [96] monitored bacterial succession of the intestine during the first four months of life of five babies, including one infant who received continuous antibiotic therapy consisting of Augmentin (a mixture of clavulanic acid and amoxicillin) for 13 days followed by Bactrimel (a mixture of trimethoprim and sulfamethoxazol) to combat urinary reflux. During the first month after birth, the universal 16S rRNA gene V6–V8 DGGE profiles of the antibiotic-treated baby were highly unstable. Main bacterial groups were identified as *E. coli* and *Enterococcus* spp., despite the fact that the administered antibiotics were expected to suppress enteric bacteria. After one month, DGGE patterns indicated the presence of a simple but remarkably stable community until the end of the study. The most significant differences between the profiles from the antibiotic-treated baby and the other four healthy babies related to the absence of *Bifidobacterium* bands in spite of a partly breast-milk diet. Likewise, a study in which nine Japanese infants were monitored during the first two months after birth demonstrated that antibiotic treatment at the beginning of life exhibits a strong influence on the establishment of a normal microbial ecosystem in the intestine [102]. Two infants who received Cefalex (a cephalosporin antibiotic) therapy in the first four days of life showed a remarkably deviating developmental pattern from the trends observed in the other nontreated subjects. DGGE analysis of V3-16S rRNA gene amplicons generated profiles with an overall low complexity that lacked bands corresponding to bifidobacteria and other strict anaerobes. In fact, band sequence analysis and random sequencing of 16S rRNA gene clone libraries indicated that *Enterobacteriaceae* were the most dominant group throughout the entire study period. In contrast to the findings of the two aforementioned studies, the SDE fingerprinting data reported by Schwiertz et al. [101] indicated that the bacterial composition in infants was not necessarily influenced by antibiotic treatment. In the latter study, the establishment and succession of the neonatal microbiota in the first month of life of 29 preterm hospitalized infants was monitored, and included seven antibiotic-treated infants receiving cefotaxime and piperazine during the first three days followed by vancomycin and amikacin therapy until inflammation was reduced which ranged up to 21 days. Overall, DGGE analysis with universal V6–V8-16S rDNA primers showed relatively stable profiles during and after antibiotic treatment, although the complexity of the banding pattern generally appeared to be lower compared to nontreated infants.

Not suprisingly, it has been shown that the human adult intestinal microbiota is affected to a different degree during antimicrobial therapy depending on the type and/or activity spectrum of the therapeutic component [77]. In the latter study, DGGE analysis of fecal samples from one patient was performed for 12 months during which different antimicrobials were administered. Visual and numerical analysis of the V3-16S rRNA gene fingerprints representing the predominant microbiota remained stable over eight months in the absence of antimicrobials (*D*_*s*_ of 88–91%) and were only minimally affected following one week ingestion of ciprofloxacin (*D*_*s*_ of 73%). In contrast, clindamycin markedly reduced the microbial complexity (*D*_*s*_ of 11–18%). However, once clindamycin therapy ceased, recovery of some intestinal groups was evident within days as indicated by the increasing similarity indices when compared to the pattern prior to antibiotic treatment (*D*_*s*_ of 36–44%). In three other patients, cefazolin (i.e., a cephalosporin with relatively low activity against intestinal anaerobes) caused only minimal alteration of V3-16S rRNA gene patterns (*D*_*s*_ of 81–83%) whereas amoxicillin/clavulanate triggered marked changes in profile compositions (*D*_*s*_ of 19–42%). Overall, the relative degree of alterations in the universal DGGE patterns tended to correspond to the relative activity spectrum of the antimicrobials against intestinal anaerobes.

In order to reduce the possible side-effects of antimicrobial therapy, probiotics are commonly administered in combination with antimicrobials during and after the period of intake [188]. In such combinatorial approaches, the absence of potentially transferable antibiotic resistance genes in the administered strain has been recognized as one of the major safety critera for human probiotics [189]. In this context, the survival and stability of probiotic strains during antimicrobial therapy are particularly relevant but have not been studied into large detail. Upon combined doxycycline (a tetracycline) and probiotic therapy, Saarela et al. [190] found that the complexity of V6–V8-16S rDNA DGGE profiles of fecal microbiota was lower (mean number of bands, 14–25) compared to those of the (control) group only taking probiotics (mean number of bands, 25–42). Probiotic strains *Lactobacillus acidophilus* LaCH-5 and *Bifidobacterium animalis* subsp. *lactis* Bb-12 from the administered commercial preparation Trevis were recovered from fecal samples, and phenotypically and genotypically characterized for their tetracycline (Tc) resistance. The Tc-susceptible strain LaCH-5 remained so during therapy, whereas recovered isolates of the Tc-resistant strain Bb-12 containing the *tet*(W) resistance gene were not found to have acquired additional Tc resistance genes. Although these observations evidence the stability of the probiotic strains as such, however, the authors did not investigate the possible effect of introducing a *tet*(W)-carrying strain during doxycycline therapy on the dissemination of this gene throughout the intestinal microbiota.

## 4. LIMITATIONS AND POTENTIAL PITFALLS

Despite its increasing use in the field of molecular microbial ecology, it is clear that SDE-based community profiling has a number of limitations that do not allow indepth analysis of microbial communities as complex as the human intestinal tract. Some of these limitations, such as detection level and taxonomic resolution, can be regarded as potential pitfalls and should be carefully taken into account during protocol development and data analysis. In fact, many of these critical factors are situated along the stages prior to the actual SDE step such as sampling and sample processing, nucleic acid extraction and community PCR, and deserve specific attention when troubleshooting SDE problems.

### 4.1. PCR bias

As discussed above, the choice of an efficient and reproducible nucleic acid extraction method ensuring optimal cell lysis and maximal removal of various PCR inhibitors present in intestinal samples is highly crucial. Likewise, possible bias introduced during PCR amplification by differential or preferential amplification of target genes from complex communities may prejudice the analysis [191]. As a result, SDE fingerprint profiles may not entirely reflect the actual composition of the predominant microbiota in the sample because of a (partial) lack of amplification of certain DNA/RNA templates. Nonproportional amplification can be due to several factors [192] including template and target sequence properties (e.g., GC-content, presence of secondary structures and template concentration) [191, 193], efficiency of primer binding influenced by primer preference, annealing temperature and primer mismatches, and the number of PCR cycles [104, 194]. Furthermore, it has also been reported that formation of chimeric and heteroduplex molecules during the amplification process [99] may generate a distorted view of the actual microbial diversity [137]. In this context, Petersen and Dahllöf [195] described a new protocol that makes use of internal standards during DNA extraction and PCR-SDE in order to compensate for experimental variability. This modification allows analyzing the relative abundance of individual species back to the original sample, thereby facilitating relative comparative analysis of diversity in complex microbial communities. Other authors have proposed to incorporate an internal standard during PCR to compare fragment staining intensities between profiles and allowing quantitative measurements of fragment intensities [75].

### 4.2. Taxonomic resolution of the 16S rRNA gene

Although every functional gene can theoretically be used, target genes for SDE fingerprinting should preferably (i) be present in a single copy in the bacterial genome; (ii) contain conserved regions among the members of the population to allow rational primer design; and (iii) comprise regions with sufficient sequence variation amongst the members of the population to produce a fingerprint revealing maximal diversity. Although the 16S rRNA gene is the prototype target in SDE applications based on the above criteria, it should be kept in mind that the possible occurrence of intraspecific multicopy operon heterogeneity [196] and the lack of a sufficient number of polymorphic regions between closely related taxa are intrinsic limitations that may affect the taxonomic resolution and complicate interpretation of SDE fingerprints. Although mostly not recognized as such, both phenomena are sources of systematic error in community fingerprinting analyses [135, 197]. As a result of the multioperon effect, a single species may appear as several bands instead of a single band in SDE profiles thereby leading to an overestimation of the diversity. For example, Satokari et al. [99] distinguished three distinct DGGE bands when analyzing the amplicon from *Bifidobacterium adolescentis* ATCC 15703^T^ obtained with *Bifidobacterium*-specific PCR primers Bif164-f and Bif662-GC-r (Table 2). Further examination revealed the presence of five rRNA gene clusters in this strain, including two clusters exhibiting microheterogeneity that were visualized as two separate bands. The third visualized band appeared to be a heteroduplex of the former two fragments. Similar observations were detected with the use of other group-specific primers targetting the *Lactobacillus* group [88] and the *Bacteroides fragilis* subgroup [64]. On the other hand, an insufficient number of polymorphic regions in the target gene may lead to an underestimation of the diversity because bands of two or more species have identical positions in the community fingerprint. For example, PCR primers Lac1 and Lac2 specific for the *Lactobacillus* group [88, Table 2] do not allow to distinguish members of the *Lactobacillus casei* group as a result of identical V3-16S rRNA gene sequences. Theoretically, the aforementioned effects can be reduced by choosing the appropriate V region in the 16S rRNA gene [131, 198]. Alternatively, single-copy housekeeping genes characterized by higher substitution rates such as *rpoB* [199–202] have recently been used as targets for microbial community profiling, but still await implementation in intestinal microbiology. To our knowledge, the use of the transaldolase gene in one study for the detection of bifidobacterial populations in fecal samples [74] is the only application in intestinal microbiology using an alternative target gene.

### 4.3. Taxonomic resolution of SDE profiles

Several authors have identified cases of comigration in SDE analysis of amplicons showing clear sequence variation [203–206]. Even for phylogenetically unrelated strains, it has been reported that the corresponding amplicons might have a similar melting behavior resulting in poor electrophoretic resolution in SDE [207–209]. The phenomenon of comigration may also cause problems to retrieve reliable sequence information from individual band extracts. To some extent, comigration can be addressed by exploiting a typical advantage of the SDE technology, that is, the use of more narrow gradients in order to produce high-resolution SDE profiles with a particular part of the original profile. This approach has been referred to as denaturing gradient gel electrophoresis gel expansion [210, DGGEGE].

Especially for SDE profiles from complex ecosystems such as the intestinal tract, band sequencing analysis may prove to be less straightforward as anticipated for several reasons. First of all, there is the possibility of multiple sequences being present in a single band due to comigration. In this case, a cloning step should be introduced prior to actual sequencing of the fragments. Furthermore, it has been reported that excised DNA fragments are commonly contaminated with ssDNA originating from other organisms present in the sample resulting in genetic contamination of the sequence profile. Elimination of the ssDNA products through mung bean or S1 nuclease treatment of the eluted DNA prior to amplification (and cloning) can increase the success rate to obtain a pure DNA sequence of the SDE band target [75, 211]. An alternative but more complex approach to overcome both aforementioned problems simultaneously involves direct cloning from the original PCR product followed by screening of individual clones against the environmental sample. In this context, it should be kept in mind that the size range of fragments that can be reliably separated by SDE is limited to 100–600 bp (optimally 200 bp). Sequence analysis of such relatively small fragments may impede reliable identification up to the species level. In addition, in silico and DGGE analysis have revealed cross-reactivity of V3- and V3–V5-16S rDNA primers with the human 18S rRNA gene [119]. Especially in case of biopsies or blood-contaminated fecal samples, coamplification of nontarget eukaryotic DNA with 16S rRNA gene primers may lead to an overestimation of bacterial biodiversity in SDE analysis when no subsequent analysis of individual community amplicons by cloning and sequencing is performed.

Next to their relative electrophoretic position and sequence composition, also the intensity and sharpness of SDE bands require special attention. Artifactual double bands, that is, the situation where each prominent band is accompanied at close distance by a second, often less intense band, have been reported in several SDE applications. Janse et al. [212] suggested that an extension of the final PCR elongation step can be sufficient to prevent the formation of artifactual second bands. The origin of double bands in SDE was explained by the authors as the formation of a secondary product due to prematurely terminated elongation during each PCR cycle. Extended incubation at high temperature during final elongation should disrupt such structures and at the same time allow the *Taq* DNA polymerase to synthesize a complete amplicon. Another observation potentially hampering the resolution of SDE analysis is linked to the phenomenon of extended fuzzy bands. The source of this inconsistency is the existence of multiple melting domains (MMDs) in the amplified fragments which results in a stepwise increase in retardation and ultimately leads to the visualization of a wide and diffuse band. Little is known about the distribution of MMD which is dependent on the target fragment and the phylogenetic group. This phenomenon has been observed when using universal 16S rRNA gene primers in SDE analysis of different types of environmental samples including feces [64], water [204], and soil [213]. Weak, fuzzy bands may erroneously be considered as background smear leading to misinterpretation of the profile richness. Curving down or smiling of bands in lanes near the edges of the gel appears to be an intrinsic feature of any SDE protocol. Although its actual cause is not entirely clear, the smiling effect is thought to result from seeping of urea and/or formamide into the buffer during the run, thereby lowering the concentration of the denaturing substances at the edges of the gel. This effect can be avoided by skipping the outer side lanes during loading and/or by applying silicone grease to the spacers [214].

### 4.4. Detection limit

The detection limit of SDE-based methods, that is, the minimum (relative) concentration or number in which any given member of a complex bacterial ecosystem needs to be present in order to be visualized in the corresponding community fingerprint, was initially estimated to approach 1% of the total population [49]. This estimation was later substantiated for TGGE analysis of intestinal samples [95], whereas Vanhoutte et al. [64] reported 10^6^ CFU/g feces (wet weight) as the detection level that could be reached by DGGE for predominant members of the fecal microbiota. In this context, it should be stressed that the detection limit is a relative value that may strongly depend on several parameters including the taxonomic complexity of the ecosystem present in the sample, the efficiency of DNA extraction, the total number of bacteria, and the relative concentration of each organism in the sample. Human stool usually contains 10^10^–10^12^ CFU/g feces, and it is thus possible that the detection limit may improve if 10^10^ CFU/g compared to 10^12^ CFU/g is present due to a lower competition among the constituting DNA templates during PCR amplification. In general, the potential to detect a specific taxon can be improved by using group-specific primers that can narrow the size of the target population. Even when using a genus-specific primer, however, the template DNA ratio may still affect the DGGE-based detection of certain species that are underrepresented in a mixed community sample [128]. Although poorly studied for SDE-based community fingerprinting of human microbiota, multiple displacement amplification (MDA) may provide another strategy to enhance the detection level especially in biopsy samples with lower bacterial counts. In MDA, the use of 3′ to 5′ exonuclease resistant random oligonucleotide primers and bacteriophage Phi29 DNA polymerase will enrich any DNA target [215], and the resulting template pool can be used for 16S rDNA PCR and subsequent SDE profiling, for example, using TTGE [216].

## 5. CONCLUSIONS AND FUTURE PERSPECTIVES

This review has highlighted the broad application spectrum of SDE-based techniques in the field of intestinal microbiology, ranging from primary assessments of the bacterial complexity and diversity of intestinal community structures to the monitoring of compositional changes at different population levels upon dietary or therapeutic interventions. In more advanced approaches, additional tools such as band sequence analysis, band position analysis, and blotting analysis permit further taxonomic exploration of the microbial communities present in the gut. Overall, SDE techniques are technically fairly simple, fast, flexible, and reproducible. Because they allow simultaneous analysis of multiple samples, SDE-based methods may be highly suitable in the selection of candidate subjects for human metagenome studies. Taken together with the ability to visualize poorly or as yet unculturable bacterial groups, these features have contributed to the current fame and reputation of SDE technology.

As is the case with any other methodology, however, also the SDE approach has a number of intrinsic limitations. Besides the general biases associated with sampling (including sample size), total DNA extraction and PCR amplification, also more specific restrictions such as intraspecies 16S rRNA gene operon heterogeneities, limited fragment length, or fuzzy bands can limit the applicability of SDE. On the other hand, it is important to always consider the significance and possible consequences of these drawbacks in the context of the study because some limitations will not be equally important when monitoring community stability compared to when assessing biodiversity. One of the most important steps in the definition of a new SDE protocol is the choice of the primer target which can already prevent several potential drawbacks related with SDE. A careful selection of the target fragment with regard to sequence variability and the distribution of multiple melting domains and a clear focus on the phylum of interest is conducive to achieve the desired resolution. In addition, also the SDE technology itself is constantly developing. Denaturing high-performance liquid chromatography (DHPLC) has relatively recently been introduced to detect genetic variation based on the SDE principle, but employs an HPLC column instead of a polyacrylamide gel matrix for amplicon separation [217]. When integrated in fully automated instruments such as the Transgenomic WAVE systems (http://www.transgenomic.com/), DHPLC analysis offers several advantages over conventional SDE analysis including the lack of gel preparation, the higher throughput, and the possibility to automatically collect sample fractions for further (sequence) analysis. DHPLC has been successfully used to analyze microbial communities with a low [218, 219] or high [220] complexity. One particular study in the field of intestinal microbiology revealed that DHPLC provides a number of technical benefits compared to DGGE but appears to have the same limitations in taxonomic resolution for profiling 16S rRNA gene amplicons [97]. Other emerging technologies such as the combination of isotopically labeled substrate analysis with RNA-DGGE [221] may offer a promising prospect for implementation in functional studies on gut microbiota.

In contemporary intestinal microbiology, SDE-based methods are rarely used as a single or end-point approach but are usually combined with culture methods and/or other molecular methods such as clone libraries, FISH, real-time PCR, and microarrays in a complementary research strategy. It is beyond doubt that these polyphasic study designs should be further pursued and developed to broaden current insights in the microbial diversity, dynamics, and interactions within the intestinal tract. In this regard, one of the major challenges ahead lies in the combined analysis of microbial presence and microbial activity. As an example of such an integrated approach, parallel DGGE analysis targetting the 16S rRNA gene as taxonomic marker and the adenosine-5′-phosphosulfate reductase subunit A gene as functional gene has been used to study the succession and diversity of sulfate-reducing bacteria in the mouse gastrointestinal tract [222]. In this regard, the wealth of information expected from large-scale sequencing efforts such as the Human Microbiome Project (http://www.nihroadmap.nih.gov/hmp/) may open new avenues for the development of SDE primers targeting specific functional genes. Considering all upcoming technological developments, it is expected that SDE community profiling will maintain and even reinforce its position in the large spectrum of molecular approaches currently employed to unravel host-microbe and micromicrobe interactions within the human microbiome. The successful incorporation of DGGE profiling in the recently launched concept of functional metagenomics [223], that is, the transgenomic characterization of key functional members of the microbiome that most influence host metabolism and hence health, brings forward a first line of evidence in that respect.

## Figures and Tables

**Table 1:** DNA extraction procedures used in SDE-based profiling of human intestinal microbial communities.

Description or reference	Sample type	Cell lysis (reagents or principle)	DNA extraction	Application(s)	Selected reference(s)
FastDNA kit (Bio101 Carlsbad, Calif, USA)^a^ FastDNA SPIN kit (Qbiogene, Carlsbad, Calif, USA)^a^	Feces; mucosa biopsies	Chemical (guanidium salts and detergents) and mechanical (bead beating using garnet mix)	Silica-based binding matrix (and spin filters)^a^	DGGE; SSCP; real-time PCR; cloning; sequencing	[43, 63, 68–76]

QIAampDNA Stool Mini Kit (Qiagen, Valencia, Calif, USA)	Feces; mucosa biopsies	Chemical (guanidium salts and detergents)	Silica-gel membrane spin columns	DGGE; TGGE; real-time PCR; cloning; sequencing	[63, 77–86]

Modified protocol of [87]	Feces	Enzymatic (lysozyme and mutanolysin) and chemical-enzymatic (SDS and proteinase K)	Phenol-chloroform-isoamylalcohol and chloroform	DGGE; sequencing	[88]

Modified protocol of [89]	Feces	Enzymatic (lysozyme and mutanolysin) and chemical (guanidiumthiocyanate-EDTA-sarkosyl)	Chloroform-isoamylalcohol	DGGE; real-time PCR	[64, 90]

[24]	Feces; mucosa biopsies	Mechanical (bead beating)	TTGE	[91–94]

[95]	Feces; cecal fluids; mucosa biopsies	Mechanical (bead beating in acid phenol)	Phenol-chloroform and chloroform	DGGE; TGGE; cloning; sequencing	[6, 65, 66, 96–102]

[32]	Feces	Mechanical-chemical (bead beating in buffer-saturated phenol and SDS)	Phenol-chloroform	Group-specific PCR; real-time PCR	[33]

[103]	Feces	Chemical (guanidiumthiocyanate and sarkosyl) and mechanical (bead beating)	Polyvinyl-polypyrrolidone	TTGE; cloning; sequencing	[21, 104–109]^b^

^a^FastDNA kit (Bio101) and FastDNA SPIN Kit (Qbiogene) only differ in the use of spin filters during the silica-DNA purification. ^b^The authors of [106–108] reported a modified protocol of [103] in which also phenol and chloroform-isoamylalcohol were applied in the extraction procedure.

**Table 2:** Universal and group-specific PCR primers used in SDE-based profiling of human intestinal microbial communities.

Target group(s)	Primer designation	Sequence (5′-3′)^a^	Target region	Selected reference(s)
Domain level				

Bacteria	HDA1^b^	ACTCCTACGGGAGGCAGCAGT	V2-V3-16S rDNA	[30, 76, 102, 110–114]
	HDA2^b^	GTATTACCGCGGCTGCTGGCAC		
	F357	CCTACGGGAGGCAGCAG	V3-16S rDNA	[64, 72, 73, 77, 79, 115–117]
	518R	ATTACCGCGGCTGCTGG		
	339F^c^	CTCCTACGGGAGGCAGCAG	V3-V4-16S rDNA	[94, 106, 107]
	788R	GGACTACCAGGGTATCTAA		
	U968-F	AACGCGAAGAACCTTAC	V6–V8-16S rDNA	[6, 64, 65, 69, 71, 79, 80, 82, 84, 85, 91, 95, 96, 101, 105, 109, 117–123]
	L1401-R	CGGTGTGTACAAGACCC		

Genus (group) level				

*Bacteroides*	FD1	AGAGTTTGATCCTGGCTCAG	16S rDNA	[124]
	RbacPre	TCACCGTTGCCGGCGTACTC		
	Bfr-F	CTGAACCAGCCAAGTAGCG	16S rDNA	[81]
	Bfr-R	CCGCAAACTTTCACAACTGACTTA		
*Bifidobacterium*	Bif164-f	GGGTGGTAATGCCGGATG	16S rDNA	[69, 79, 82, 85, 99, 101, 105, 108, 118, 125–127]
	Bif662-r	CCACCGTTACACCGGGAA		
	g-Bifid-F	CTCCTGGAAACGGGTGG	16S rDNA	[64]
	g-Bifid-R	GGTGTTCTTCCCGATATCTACA		
	ForTal	CGTCGCCTTCTTCTTCGTCTC	transaldolase gene	[74]
	RevTal	CTTCTCCGGCATGGTGTTGAC		
*Helicobacter*	658f	TGGGAGAGGTAGGTGGAAT	16S rDNA	[128]
	1067R	GCCGTGCAGCACCTGTTTTCA		
*Enterococcus*	Ent1017F	CCTTTGACCACTCTAGAG	16S rDNA	[64]
	Ent1263R	CTTAGCCTCGCGACT		
*Lactobacillus* group^d^	Lac1	AGCAGTAGGGAATCTTCCA	16S rDNA	[64, 86, 88, 125, 127, 129, 130]
	Lac2	ATTYCACCGCTACACATG		
	27f (also Bact-0011f)	AGAGTTTGAT(C/T)(A/C)TGGCTCAG	16S rDNA	[79, 98, 118]
	Lab-0677r	CACCGCTACACATGGAG		
	Lab-0159f	GGAAACAG(A/G)TGCTAATACCG	16S rDNA	[98, 118]
	Uni-0515-r	ATCGTATTACCGCGGCTGCTGGCA		
	Lab-0159f	GGAAACAG(A/G)TGCTAATACCG	16S rDNA	[124]
	Lab-0677r	CACCGCTACACATGGAG		

Species group level				

*Bacteroides fragilis* subgroup^e^	g-Bact-F	ATAGCCTTTCGAAAGRAAGAT	16S rDNA	[73]
	g-Bact-R	CCAGTATCAACTGCAATTTTA		
	Bact 596F	TCAGTTGTGAAAGTTTGCG	16S rDNA	[64]
	Bact 826R	GTRTATCGCMAACAGCGA		
	Bact 531F	ATACGGAGGATCCGAGCGTTA	16S rDNA	[90]
	Bact 766R	CTGTTTGATACCCACACT		
*Clostridium* phylogenetic clusters XI and XIVa^f^	Erec 688F	GCGTAGATATTAGGAGGAAC	16S rDNA	[90]
	Erec 841R	TGCGTTWGCKRCGGCACCG		

^a^A GC-clamp is attached to the 5′ end of either the forward or reverse primer. ^b^Primers HDA1 and HDA2 have the same core sequence as primers 341 f and 518 r, respectively, but with a few additional nucleotides at both 5′ and 3′ ends. ^c^Primer 339f has the same core sequence as primer 341 f but with two additional nucleotides at the 5′ end. ^d^The *Lactobacillus* group comprising the genera *Lactobacillus, Leuconostoc, Pediococcus, Weissella*, and *Aerococcus* (the latter genus was originally not described as target of the Lac1/2 primers). ^e^The *Bacteroides fragilis* subgroup comprising *B. fragilis, B. acidifaciens, B. caccae, B. eggerthii, B. ovatus, B. stercoris, B. thetaiotaomicron, B. uniformis*, and *B. vulgatus*. ^f^ *Clostridium* phylogenetic cluster XI represents the *Clostridium lituseburense* group, whereas *Clostridium* phylogenetic cluster XIVa represents the *Clostridium coccoides-Eubacterium rectale* group.

**Table 3:** Selection of dietary intervention studies using SDE-based community fingerprinting.

Component^a^	Administered component	Reference(s)^b^
p	Levan-type exopolysaccharides, levan, inulin and FOS	[111]
p	GOS and FOS	[75]
p	Difructose anhydride III (DFA III)	[72, 73]
P	*Lactobacillus rhamnosus* DR20	[76, 88]
P	*Lactobacillus paracasei* F19	[98]
P	VSL#3^®^ (probiotic mixture of eight strains)	[110]
P	*Bifidobacterium longum* (Bifina^®^) and yogurt with *Bifidobacterium animalis* DN-173 010	[179]
y	*Lactobacillus delbrueckii* subsp. *bulgaricus* and *Streptococcus thermophilus*	[68]
p, P	Inulin or *B. longum* (Bifina^®^)	[180]
pP	Inulin-containing probiotic yogurts	[127]
pP	GOS-containing probiotic yogurt	[69]
p, P, pP	GOS and/or *Bifidobacterium lactis* Bb-12	[100]
p, P, pP	Lactulose and/or *Saccharomyces boulardii*	[90]
o	Black tea	[120]
o, op, oP	Isoflavones and FOS or *B. animalis* DN-173 010	[105]

^a^p: prebiotic; P: probiotic; pP: synbiotic; y, yogurt; o: other. ^b^All studies used DGGE as SDE method except in [105], where TTGE was used.
